# Supplementary material for: Mobility enhancement in heavily doped semiconductors via electron cloaking
Source: Nat Commun. 2022 May 6;13:2482. doi: 10.1038/s41467-022-29958-2 (PMC9076901; doi:10.1038/s41467-022-29958-2)
Supplement: Supplementary file 1 — Supplementary Information [file 41467_2022_29958_MOESM1_ESM.pdf]

Supplementary Information

Mobility enhancement in heavily doped semiconductors via electron cloaking

Jiawei Zhou[1], Hangtian Zhu[2], Qichen Song[1], Zhiwei Ding[1], Jun Mao[2], Zhifeng Ren[2], Gang Chen[1]

[1]*Department of Mechanical Engineering, Massachusetts Institute of Technology, Cambridge, MA 02139, USA*

[2] *Department of Physics and Texas Center for Superconductivity, University of Houston, Houston, TX 77204, USA*

Supplementary Note

Model study of defect scattering

Figure 1 of the manuscript showed our electron transport calculation results for a model semiconductor, which illustrates the general effect of electron-defect interaction on electron transport, and demonstrates the possibility of achieving ideal electron cloaking (Fig. 1c,e,g). The electron mobility is calculated as[1]

$$\mu_e = \left[\frac{N_v e}{3}\int v^2 \tau \left(-\frac{\partial f^0}{\partial E}\right) D(E)\mathrm{d}E\right]/n \quad (1)$$

Here the integration spans over electron states close to the Fermi level, $N_v = 6$ is the band degeneracy and e is the electronic charge. E is the electron energy. v is the electron group velocity and is related to the conductivity effective mass $m_{eff,c}$ (0.268 m_e, where m_e is the free electron mass) *via* $v^2 = 2E/m_{eff,c}$. τ is the electron relaxation time. $f^0 = 1/(1+\exp(\frac{E-\mu}{k_B T}))$ is the Fermi-Dirac distribution function with μ being the Fermi level. $D(E)$ is the electronic density of states, related to the density-of-states effective mass $m_{eff,DOS}$ (0.33 m_e) via $D = (\frac{2m_{eff,DOS}}{\hbar^2})^{3/2}\frac{\sqrt{E}}{2\pi^2}$ with $\hbar$ being the reduced Planck constant. n is the carrier concentration.

The electron relaxation time τ, the inverse of the scattering rate, is determined via Matthiessen's rule considering both intrinsic electron-phonon interactions and extrinsic electron-defect interactions: $1/\tau = 1/\tau_{e-ph} + 1/\tau_{e-d}$. The intrinsic electron-phonon interactions consider both acoustic phonon and optical phonon scatterings *via* corresponding deformation potentials[1]:

$$\frac{1}{\tau_{e-ph,acoustic}} = \frac{\pi D_A^2 k_B T}{\hbar C_l} D(E) \quad (2)$$

where $D_A = 9.6$ eV is the acoustic deformation potential and $C_l = 190.7$ GPa is the elastic constant, and

$$\frac{1}{\tau_{e-ph,optical}} = (N_v - 1)\frac{\pi D_O^2}{2\rho\omega_O}\left[\frac{1}{e^{\frac{\hbar\omega_O}{k_B T}}-1} D(E+\hbar\omega_O) + \left(\frac{1}{e^{\frac{\hbar\omega_O}{k_B T}}-1}+1\right) D(E-\hbar\omega_O)\right] \quad (3)$$

where $D_O = 6.5$ eV/Å is the optical deformation potential, $\rho = 2330$ kg/m^3 is the material density, and ω_O is the angular frequency of the optical phonon ($\omega_O = 2\pi f_O$, with $f_O = 472$ cm^{-1}). The sum of these two scattering rates leads to the intrinsic electron-phonon scattering rates: $1/\tau_{e-ph} = 1/\tau_{e-ph,acoustic} + 1/\tau_{e-ph,optical}$.

The electron-defect scattering rate $1/\tau_{e-d}$ is calculated based on the partial wave analysis which evaluates the scattering of electrons by a spherically symmetric potential[2]

$$\frac{1}{\tau_{e-d}} = N_d \frac{4\pi}{\hbar^2}\frac{1}{m_{eff,DOS}\sqrt{2m_{eff,DOS}E}}\sum_{l=0}^{\infty}(l+1)\sin^2(\delta_l - \delta_{l+1}) \quad (4)$$

where N_d is the volume density of the defect and δ_l is the phase shift of the electron wave with quantum number l. The maximum number of l in the summation is taken to be 10, which is found to be sufficient to achieve convergence. The scattering of electron waves is obtained by solving the Schrödinger equation. For spherically symmetric potential V, the wavefunction takes the form of $\Psi(\boldsymbol{r}) = \sum_{l,m} a_{l,m} \frac{u_l(r)}{r} Y_l^m(\theta, \phi)$, where $a_{l,m}$ are constants, and $Y_l^m(\theta, \phi)$ are the normalized spherical harmonics. The radial function $u_l(r)$ satisfies[2]

$$\frac{d^2 u_l}{dx^2} + \left[\frac{2ma_0^2 E}{\hbar^2} - \frac{2ma_0^2 V(r)}{\hbar^2} - \frac{l(l+1)}{x^2}\right] u_l = 0 \tag{5}$$

where the coordinate has been re-scaled by a characteristic length a_0 ($x = \mathrm{r}/a_0$). Two forms of defect potential have been considered. The first corresponds to a pure Coulomb potential

$$\Delta\hat{V} = -\frac{e^2}{4\pi\varepsilon\varepsilon_0 r} \tag{6}$$

where $\varepsilon = 11.7$ is the dielectric constant, and ε_0 is the vacuum permittivity. The second corresponds to a more practical case in which the central part is replaced with a central potential

$$\Delta\hat{V} = \begin{cases} V_0 & r \le r_0 \\ -\frac{e^2}{4\pi\varepsilon\varepsilon_0 r} & r > r_0 \end{cases} \tag{7}$$

with $r_0 = 1.6$ Å characterizing the range of the short-range potential, and where $V_0 = 9.7$ eV is the short-range potential energy. The solution to Eq. (5) is obtained by numerical integration based on the Gauss-Jackson method[3]. The phase shift is determined by matching the obtained solution to the expected asymptotic form of $u_l \approx xB_l \cos(\delta_l)\left[j_l\left(\sqrt{\frac{2ma_0^2E}{\hbar^2}}x\right) - \tan(\delta_l) y_l\left(\sqrt{\frac{2ma_0^2E}{\hbar^2}}x\right)\right]$, where $j_l(z)$ and $y_l(z)$ are the regular and irregular spherical Bessel function of order l, respectively. Once the phase shifts are determined, the electron-defect scattering rates can be readily computed using Eq. (4).

Extraction of central cell defect potential

The impurity potential is defined as the difference in the total electronic potential from first-principles calculations between the system with the defect and the original pristine system $\Delta\hat{V} = V_d - V_{bulk}$. One typically builds a large supercell and calculates the defect potential for the pure and defected systems separately. However, as the supercell size is often limited to be no more than a few lattice vectors long, the long-range Coulomb potential is cut off at the supercell boundary and cannot be correctly represented. The challenge of the electron-defect scattering calculation is thus to obtain a correct full profile of the defect potential including both its long-range and short-range parts.

While the central cell potential can vary significantly with the defect and lattice type, we recognize that the long-range portion of the defect potential can be well described by an analytic Coulomb potential profile. This fact has been utilized to correct the defect formation energy in finite size supercell calculations[4]. Here we use this fact to recover the short-range part of the defect potential, which essentially is the central cell potential. To illustrate this, we consider n-type silicon as an example. We built a silicon supercell with a $3 \times 3 \times 3$ conventional unit cell and replaced one silicon atom with a dopant atom (phosphorous, arsenic, or antimony). The defect potential is obtained by subtracting the potential of pure silicon from the one with the dopant atom. For the latter calculation, the total charge of the supercell is taken to be $+1e$, making the dopant positively charged (corresponding to n-type). The defect potentials corresponding to the different dopants in silicon are shown in Fig. S3a.

If the defect potential is short-ranged, the potential profile far away from the defect atom (corresponding to the middle point in the plot) should be flat. However, it is clear from Fig. S3a that while different dopants show different short-range profiles, all exhibit a gradually decaying profile far away from the defect. This gradually decaying profile is due to the long-range Coulomb potential of the defect charge. If we consider the Coulomb potential of an infinite periodic array of charge Ze at locations $\boldsymbol{R}_i$ corresponding to the corners of the periodic supercell[5], the Coulomb potential at $\boldsymbol{r}$ is

$$\Delta\hat{V}_{lr} = -\sum_i \frac{Ze^2}{\sqrt{|\bar{\varepsilon}|}} \frac{\mathrm{erfc}\left(\gamma\sqrt{(\boldsymbol{R}_i-\boldsymbol{r})\cdot\bar{\varepsilon}^{-1}\cdot(\boldsymbol{R}_i-\boldsymbol{r})}\right)}{\sqrt{(\boldsymbol{R}_i-\boldsymbol{r})\cdot\bar{\varepsilon}^{-1}\cdot(\boldsymbol{R}_i-\boldsymbol{r})}} - \sum_{\boldsymbol{G}_i}^{i\neq 0} \frac{4\pi Ze^2}{\Omega} \frac{\exp\left(-\boldsymbol{G}_i\cdot\bar{\varepsilon}\cdot\frac{\boldsymbol{G}_i}{4\gamma^2}\right)}{\boldsymbol{G}_i\cdot\bar{\varepsilon}\cdot\boldsymbol{G}_i} \exp(i\boldsymbol{G}_i\cdot\boldsymbol{r}) + \frac{\pi Ze^2}{\Omega\gamma^2} \qquad (8)$$

Here $\bar{\varepsilon}$ is the dielectric tensor computed from first principles, Ω is the supercell volume, $Z = 1$ is the defect charge, and γ is a convergence parameter for the Ewald summation. This long-range Coulomb potential is also plotted in Fig. S3a as a reference and matches well with the asymptotic trend of all defect potentials extracted from first principles. If we subtract this long-range term from the defect potential, we are then left with only a short-range component (Fig. S3b), which becomes flat away from the defect. In Fig. S3b we have also aligned the potential at the farthest distance from the defect to zero, based on its short-range nature. This shows that the above procedure enabled the extraction of the short-range defect potential from the finite-size supercell calculations. The effects of long-range Coulomb potential on electron-defect interactions can then be later added into the scattering matrix *via* an analytic expression, as explained in detail in Methods.

First-principles electron transport calculation

First-principles calculations of electron transport properties (specifically, the electrical conductivity σ and the Seebeck coefficient S) are based on the Boltzmann transport theory[1]:

$$\begin{cases} \sigma = \frac{e^2}{3\Omega_0 N_{\boldsymbol{k}}} \sum_{\boldsymbol{k}\alpha} v_{\boldsymbol{k}\alpha}^2 \tau_{\boldsymbol{k}\alpha} \left(-\frac{\partial f_{\boldsymbol{k}\alpha}^0}{\partial E}\right) \\ S = \frac{e}{3\sigma\Omega_0 N_{\boldsymbol{k}} T} \sum_{\boldsymbol{k}\alpha} (E-\mu) v_{\boldsymbol{k}\alpha}^2 \tau_{\boldsymbol{k}\alpha} \left(-\frac{\partial f_{\boldsymbol{k}\alpha}^0}{\partial E}\right) \end{cases} \qquad (9)$$

where e is the electronic charge, Ω_0 is the unit cell volume, $N_{\boldsymbol{k}}$ is the number of $\boldsymbol{k}$ points, α is the band index, $v_{\boldsymbol{k}\alpha}$ is the electron group velocity, $\tau_{\boldsymbol{k}\alpha}$ is the electron relaxation time, E is the electron energy, μ is the Fermi level, and $f_{\boldsymbol{k}\alpha}^0$ is the Fermi-Dirac distribution. The electron energy and group velocity are derived from the electronic band structure. The equilibrium properties of electrons of half-Heusler materials are calculated from first principles using the QUANTUM ESPRESSO software package[6]. We use the generalized gradient approximation (GGA) of Perdew, Burke and Ernzerhof with the Troullier-Martins-type norm-conserving semilocal pseudopotential[7] (corresponding to pbe-mt.UPF in the QUANTUM ESPRESSO pseudopotential library). A cutoff energy of 120 Ryd and a $6 \times 6 \times 6$ **k**-mesh are used to determine the equilibrium lattice constant. The equilibrium properties of phonons and the electron-phonon interaction matrices are calculated *via* density functional perturbation theory[8] for a $6 \times 6 \times 6$ **q**-mesh (with a $6 \times 6 \times 6$ **k**-mesh for the electron-phonon interaction matrix). We then use the EPW software package[9] to interpolate the electronic information and the phonon information, as well as the electron-phonon coupling matrices to a fine mesh. A fine mesh is required to ensure that the calculation of transport properties based on Eq. (9) is converged. In Eq. (9), the electron relaxation time is determined *via* Matthiessen's rule considering both intrinsic electron-phonon scattering rates and extrinsic electron-defect scattering rates: $1/\tau = 1/\tau_{e-ph} + 1/\tau_{e-d}$. The intrinsic electron-phonon scattering rates are related to the electron-phonon interaction matrix $g(\boldsymbol{k}, \boldsymbol{k}+\boldsymbol{q}, \boldsymbol{q})$ via[10]

$$\frac{1}{\tau_{\boldsymbol{k}}^{e-ph}} = \frac{2\pi}{\hbar}\frac{1}{N_{\boldsymbol{q}}}\sum_{\boldsymbol{q}} |g(\boldsymbol{k}, \boldsymbol{k}+\boldsymbol{q}, \boldsymbol{q})|^2 \cdot \begin{bmatrix} (n_{\boldsymbol{q}} + f_{\boldsymbol{k}+\boldsymbol{q}})\delta(E_{\boldsymbol{k}} - E_{\boldsymbol{k}+\boldsymbol{q}} + \hbar\omega_{\boldsymbol{q}}) \\ +(n_{\boldsymbol{q}} + 1 - f_{\boldsymbol{k}+\boldsymbol{q}})\delta(E_{\boldsymbol{k}} - E_{\boldsymbol{k}+\boldsymbol{q}} - \hbar\omega_{\boldsymbol{q}}) \end{bmatrix} \quad (10)$$

which sums over all possible scattering processes that satisfy momentum and energy conservations using a tetrahedral integration method, where $N_{\boldsymbol{q}}$ is the number of **q** points, $n_{\boldsymbol{q}}$ is the Bose-Einstein distribution for phonons, and the delta functions indicate the energy conservation. The electron-defect scattering rates are determined by the electron-defect interaction matrix $g_{e-d}(\boldsymbol{k}, \boldsymbol{k'})$ via

$$\frac{1}{\tau_{\boldsymbol{k}}^{e-d}} = N_d \Omega_0 \frac{2\pi}{\hbar}\frac{1}{N_{\boldsymbol{k}}}\sum_{\boldsymbol{k'}} \left(1 - \frac{\mathrm{v}_{\boldsymbol{k}} \cdot \mathrm{v}_{\boldsymbol{k'}}}{|\mathrm{v}_{\boldsymbol{k}}||\mathrm{v}_{\boldsymbol{k'}}|}\right) |g_{e-d}(\boldsymbol{k}, \boldsymbol{k'})|^2 \delta(E_{\boldsymbol{k}} - E_{\boldsymbol{k'}}) \quad (11)$$

where N_d is the volume density of defects. The calculation of $g_{e-d}(\boldsymbol{k}, \boldsymbol{k'})$ has been detailed in Methods. By adding all scattering rates together, we obtain the total electron scattering rates, which are then inserted into Eq. (9) to yield the electron transport properties.

Spatial projection of electron-defect interaction

While in the main text we mainly discussed the defect potential and its trend in the periodic table based on the ionic radius, the actual electron-defect interaction strength is governed by the electron-defect interaction matrix, which is a spatial product of the defect potential and the electronic wavefunctions

$$\langle \psi_{\boldsymbol{k'}} | \Delta\hat{V} | \psi_{\boldsymbol{k}} \rangle = \int d\boldsymbol{r}\, \psi_{\boldsymbol{k'}}^*(\boldsymbol{r}) \Delta\hat{V}(\boldsymbol{r}) \psi_{\boldsymbol{k}}(\boldsymbol{r}) \quad (12)$$

Usually, the electronic wavefunction can be approximately expressed by a linear combination of atomic orbitals on different atomic sites

$$\psi_{\boldsymbol{k}}(\boldsymbol{r}) = e^{i\boldsymbol{k}\cdot\boldsymbol{r}} \sum c_{\alpha i} \phi_\alpha^i(\boldsymbol{r}) \tag{13}$$

where ϕ_α^i denotes the i-th atomic orbital on atom α and has significant non-zero values only around atom α. With this, we have

$$\langle \psi_{\boldsymbol{k}'} | \Delta\hat{V} | \psi_{\boldsymbol{k}} \rangle = e^{i(\boldsymbol{k}-\boldsymbol{k}')\cdot\boldsymbol{r}} \sum_{\alpha i,\beta j} c_{\alpha i} c_{\beta j}^* \int d\boldsymbol{r}\, {\phi_\beta^j}^*(\boldsymbol{r}) \Delta\hat{V}(\boldsymbol{r}) \phi_\alpha^i(\boldsymbol{r}) \tag{14}$$

We denote the defect site as γ. Because the defect potential $\Delta\hat{V}(\boldsymbol{r})$ is significant only around the defect site, it is then clear that in addition to the magnitude of the defect potential, the electron-defect interaction also depends on the projection of wavefunctions on given atomic sites, which are described by the pre-factors $c_{\alpha i} c_{\beta j}^*$. For the same defect potential, wavefunctions with larger projected density-of-states on the defect site (namely large non-zero values of $c_{\gamma i}$) will lead to stronger electron-defect interactions, and vice versa.

Supplementary Figure

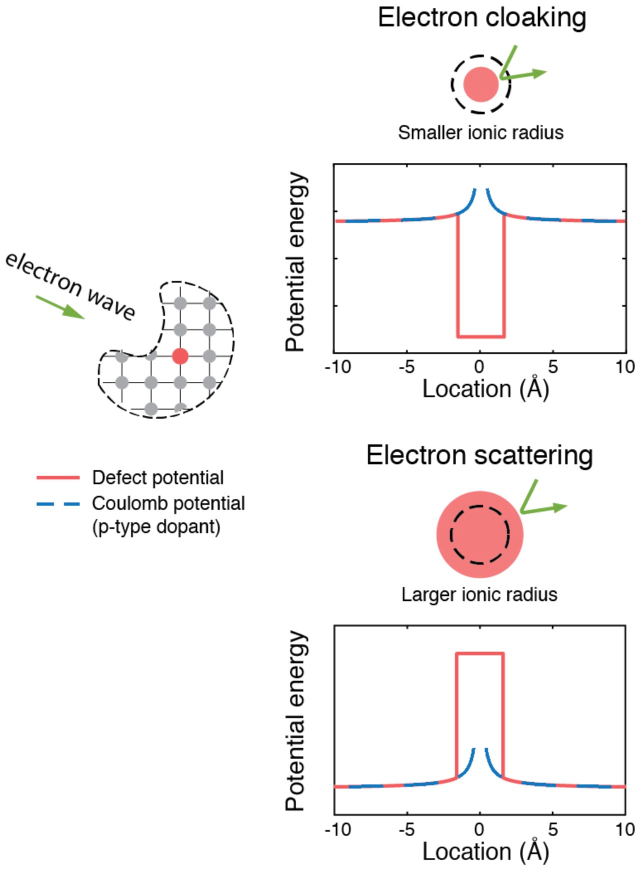

Figure S1. **Illustration of a propagating electron wave scattered by the perturbed electronic potential due to the presence of a charged p-type dopant**. For a p-type dopant, the long-range Coulomb potential is repulsive to electrons (contrary to the n-type case). In this case, if the dopant atom has a smaller ionic radius than the host atom, the perturbation tends to create an attractive force for electrons, which counteracts the Coulomb potential (electron-cloaking scenario). On the other hand, if the dopant atom has a larger ionic radius, it tends to create a repulsive force, which then adds to the long-range Coulomb potential (electron-scattering scenario).

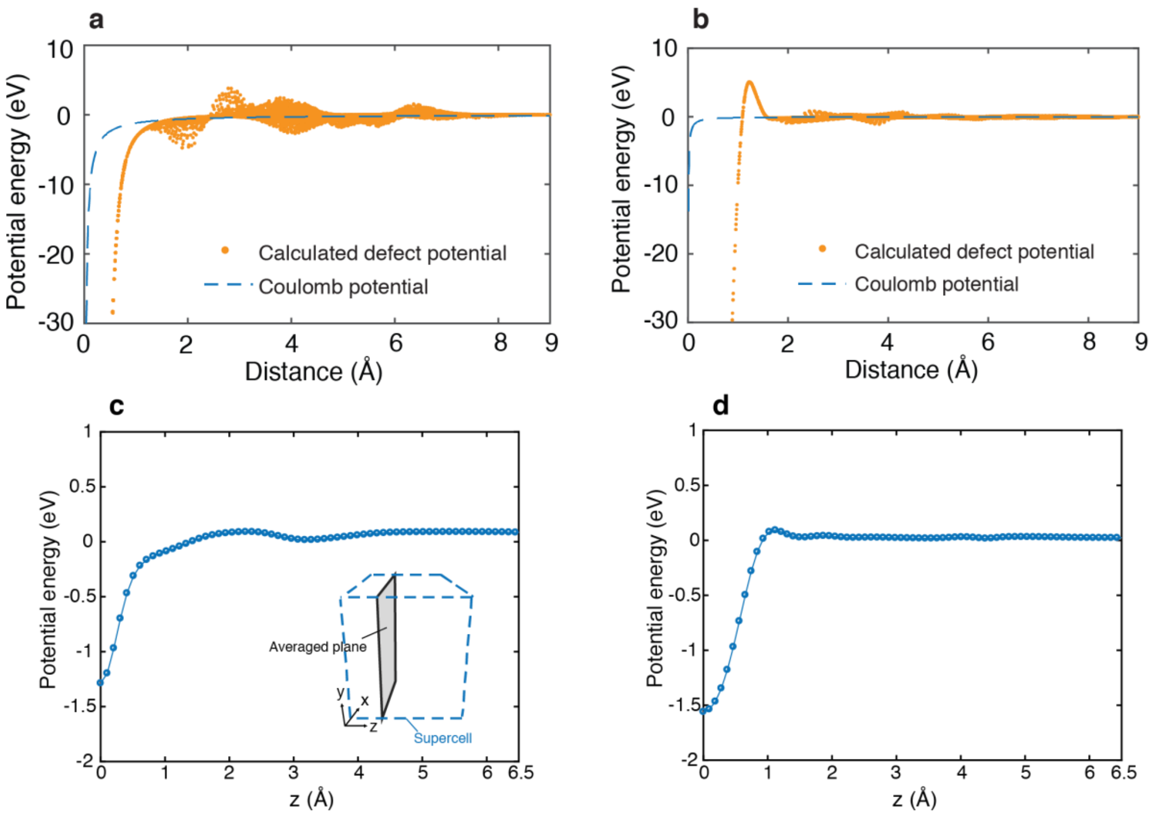

Figure S2. **Defect potential extracted from first principles calculations**. **a-b**, Directly extracted defect potentials for (**a**) Si-doped GaAs, and (**b**) Bi-doped PbTe, plotted from the location of the defect. In each case, the long-range part can be approximately described by the Coulomb potential. The distortions observed in the range between ~2 Å and ~7 Å are due to relaxation of atomic positions in the presence of the defect. Significant deviation from the Coulomb potential can be seen at short range (< 1 Å). The asymptotic trend given by the Coulomb field is recovered as one moves away from the defect. **c-d**, Planar averaged defect potentials for Si-doped GaAs (**c**) and Bi-doped PbTe (**d**). The planar averaged potential is calculated by $\overline{\Delta V}(z) = \frac{1}{A}\iint_0^a dxdy\Delta\hat{V}(x,y,z)$ where a is the lattice vector length of the supercell, and A is the area of the x-y plane of the supercell, as indicated by the inset of (**c**). The z axis starts from the plane containing the defect.

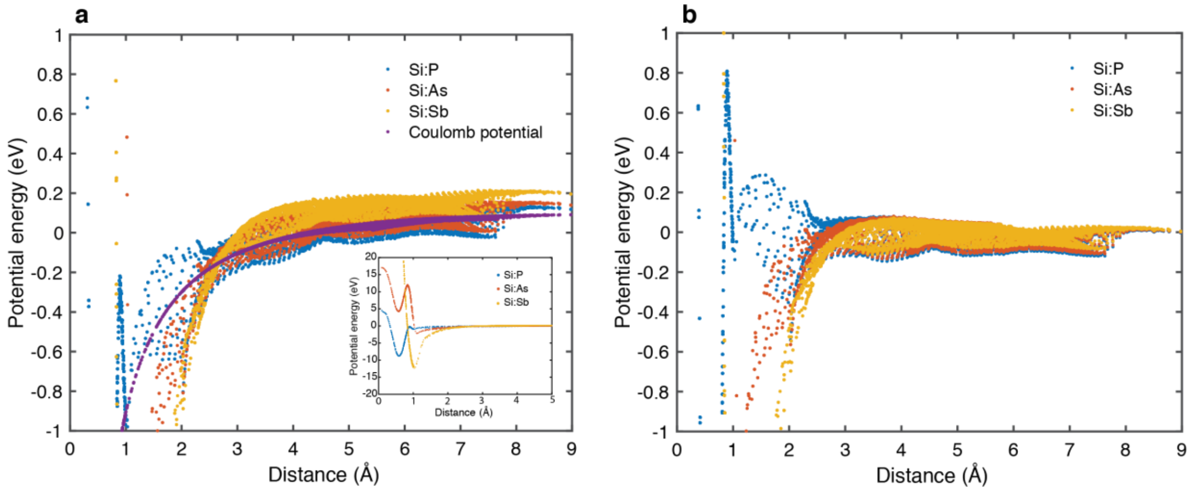

Figure S3. **Illustration of subtraction of the long-range Coulomb potential from *ab initio* defect potential profiles**. **a**, Uncorrected defect potentials for silicon doped with different n-type dopants (P, As, or Sb, as indicated in the figure legend) directly extracted from first-principles calculations, compared with the analytic long-range Coulomb potential calculated based on Eq. (8) in Supplementary Note. The three-dimensional defect potentials are projected as a function of the distance from the defect, located at the center of the supercell. The asymptotic trend given by the Coulomb field is recovered as one moves away from the defect. The inset shows the short-range parts with a larger energy scale. **b**, Corrected defect potentials, showing their short-range nature. Here, in calculating the defect potential, we have ignored the atomic relaxation to emphasize the long-range decaying part. The potentials at the farthest location away from the defect have also been aligned to zero.

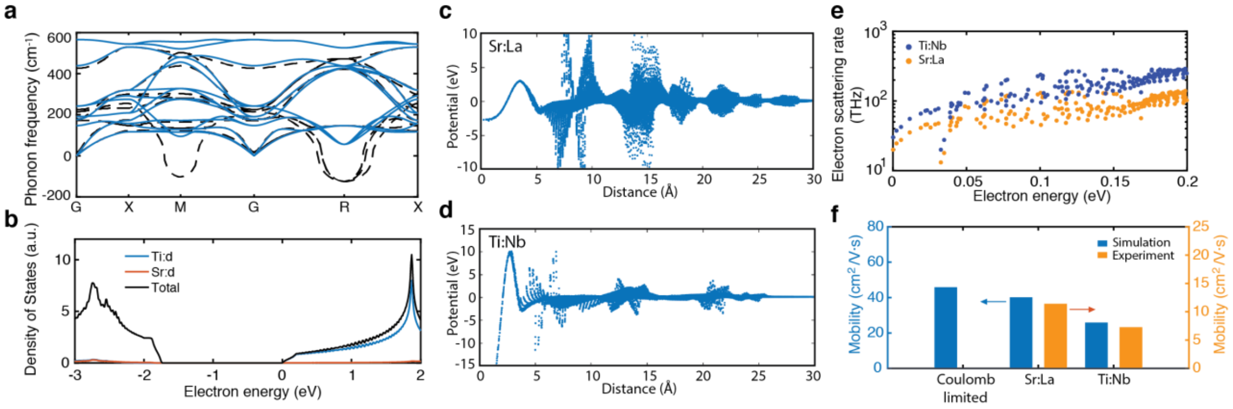

Figure S4. Electron transport simulation of $SrTiO_3$. a, Computed phonon dispersion of $SrTiO_3$. Dashed lines are results from density functional perturbation theory, which gives rise to imaginary phonon frequencies. The solid lines are obtained by fitting force constants to a force-displacement dataset from *ab initio* molecular dynamics study[11] using VASP package. The fitting was performed using ALAMODE package[12]. The latter method correctly reproduces the stable phonon modes at 300 K. **b**, Projected electronic density-of-states near the band edge, showing that electron states near the conduction band edge have dominant contributions from *d* orbitals on Sr atom. **c-d**, Defect potentials for (**b**) La dopant on Sr site, and (**c**) Nb dopant on Ti site. **e**, Comparison of electron-defect scattering rates for La and Nb dopants at the carrier concentration of 6×10^{19} cm^{-3}. The defect potential includes both the short-range perturbation and long-range Coulomb potential. **f**, Mobilities considering different scattering conditions (Coulomb scattering only, and those that consider the full defect potential corresponding to La and Nb dopants), and comparison between simulation and experiment[13]. The calculated mobilities assume a carrier concentration of 6×10^{19} cm^{-3}.

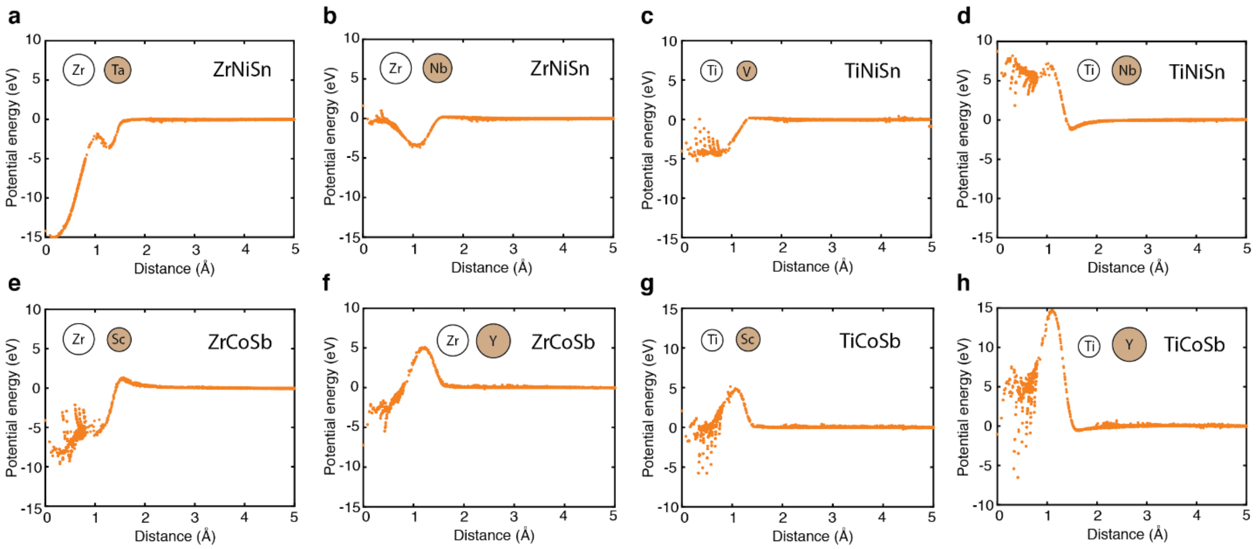

Figure S5. Calculated defect potentials for selected dopant/host pairs. Results are shown for n-type Ta-doped ZrNiSn (**a**), Nb-doped ZrNiSn (**b**), V-doped TiNiSn (**c**), Nb-doped TiNiSn (**d**), and p-type Sc-doped ZrCoSb (**e**), Y-doped ZrCoSb (**f**), Sc-doped TiCoSb (**g**), and Y-doped TiCoSb (**h**). The ionic sizes are drawn in the inset (same as Figure 3a). Here, in presenting the defect potential, we have ignored the atomic relaxation to focus on the short-range defect potential.

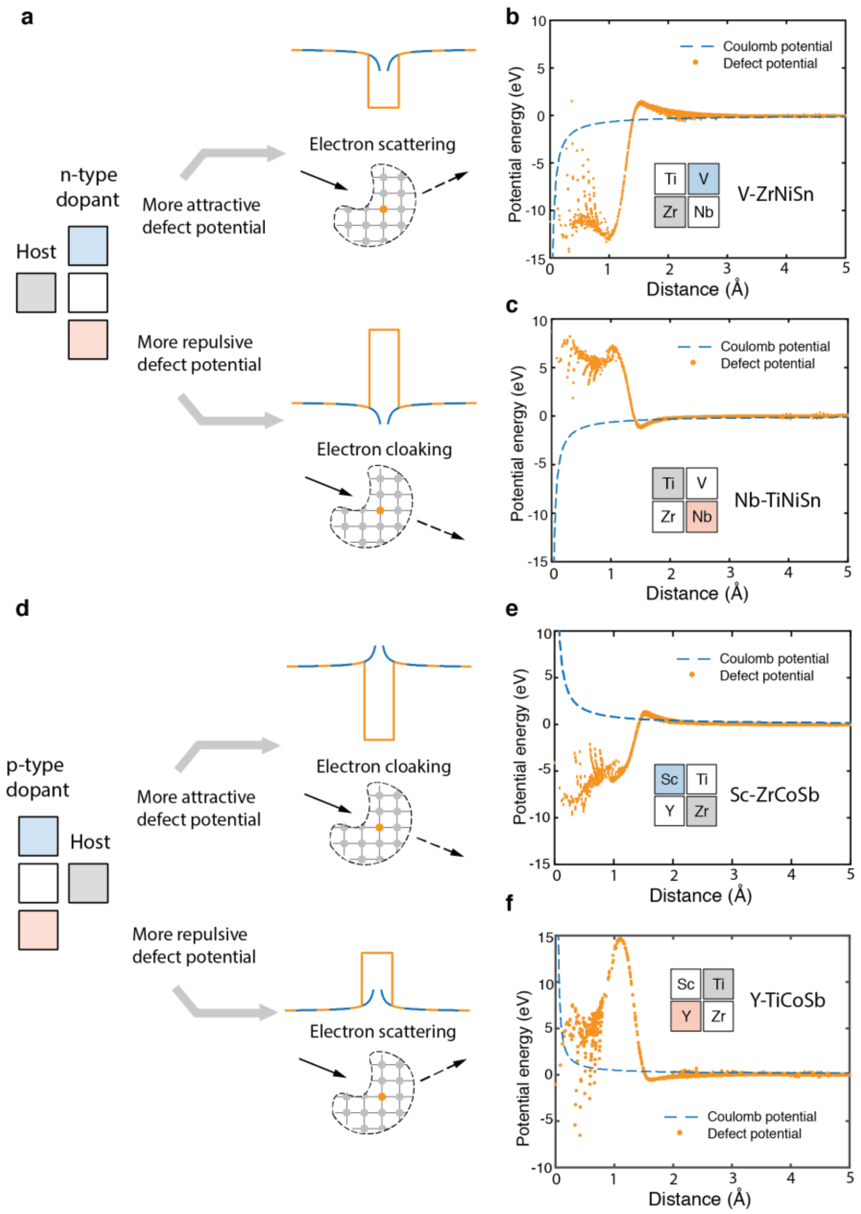

Figure S6. **Illustrations of defect potentials in n-type and p-type materials leading to electron-scattering and electron-cloaking scenarios**. **a**, The electron-cloaking effect is achieved in n-type material when the Coulomb potential is counterbalanced by a repulsive central cell potential from a dopant. **b-c**, Calculated defect potential for n-type (**b**) V-doped ZrNiSn (electron-scattering scenario) and (**c**) Nb-doped TiNiSn (electron-cloaking scenario). **d**, The electron-cloaking effect is achieved in p-type material when the Coulomb potential is counterbalanced by an attractive central cell potential from a dopant. **e-f**, Calculated defect potential for p-type (**e**) Sc-doped ZrCoSb (electron-cloaking scenario) and (**f**) Y-doped TiCoSb (electron-scattering scenario). Here, in presenting the defect potential, we have ignored the atomic relaxation to focus on the short-range defect potential. Coulomb potentials are also plotted in (**b-c**) and (**e-f**), to show that the asymptotic trend given by the Coulomb field is recovered as one moves away from the defect.

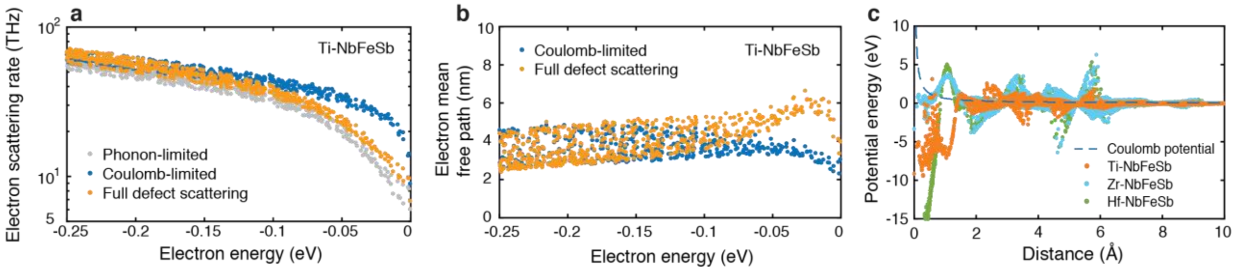

Figure S7. **Electron transport details in p-type NbFeSb**. **a**, Scattering rates for holes in p-type Ti-doped NbFeSb, showing that the short-range defect potential leads to a significant reduction in the scattering rates compared to those limited by Coulomb scattering. The energy is relative to the valence band edge. **b**, Charge carrier mean free paths as a function of energy in Ti-doped NbFeSb at a carrier concentration of 2×10^{20} cm^{-3}. While Coulomb scatterings severely limit the mean free paths, the central cell potential can counteract this effect and increase the carrier mean free paths by almost a factor of two near the band edge. **c**, Calculated defect potential of p-type dopants (Ti, Zr, and Hf) in NbFeSb. The larger power factors obtained with Ti and Hf dopants can be understood based on their attractive short-range potential which counterbalances the long-range Coulomb scatterings.

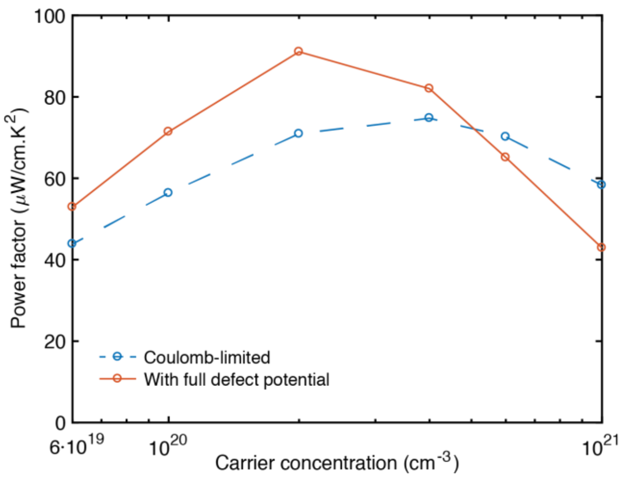

Figure S8. **Thermoelectric power factor of p-type Ti-doped TaFeSb at room temperature**. Both curves consider intrinsic electron-phonon interactions. The dashed curve includes electron scatterings by the Coulomb potential, while the solid curve considers electron scatterings by the full defect potential. Due to the counteraction between the strong central cell potential of the Ti dopant and the Coulomb potential, the full defect potential leads to an enhanced optimal power factor.

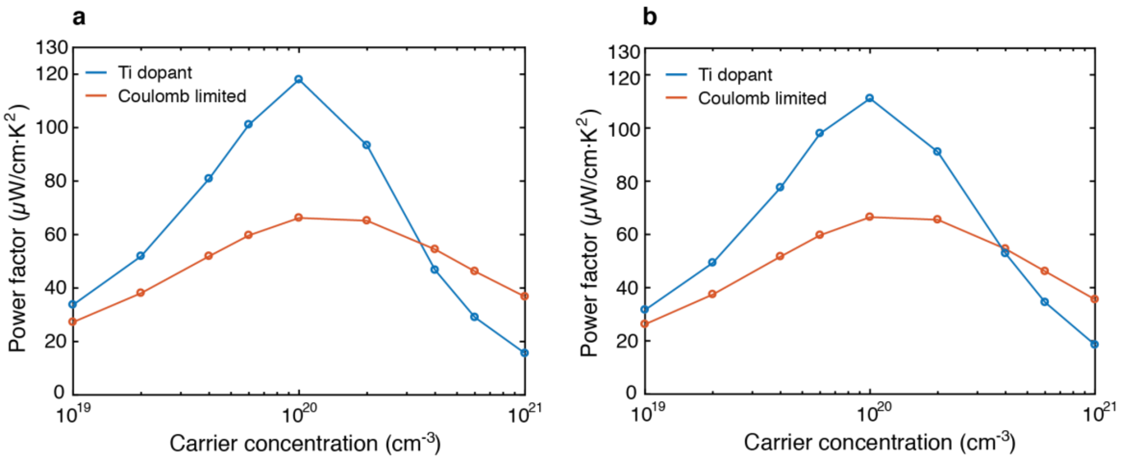

Figure S9. **Thermoelectric power factor at 150 K**. **a**, p-type Ti-doped NbFeSb. **b**, p-type Ti-doped TaFeSb. For both plots, the red curve only considers electron scatterings by phonons and by the Coulomb potential, while the blue curve considers electron scatterings by phonons and the full defect potential (including the short-range part). Due to the counteraction between the strong central cell potential of the Ti dopant and the Coulomb potential, a larger enhancement in power factor is observed at lower temperatures.

Table S1. Dielectric constants of representative oxides and thermoelectric materials.

Material	Dielectric constant (relative)
ZrO_2	29[14]
HfO_2	25[14]
Ta_2O_5	26[14]
La_2O_3	30[14]
$LaAlO_3$	30[14]
Nb_2O_5	35[14]
TiO_2	95[14]
Bi_2Te_3	290 (∥, 15K)[15]
PbTe	414[15]
SnSe	42 (c axis), 45 (a axis)[15]
NbFeSb	45[16]
Mg_3Sb_2	32[16]

Reference

1. Lundstrom, M. *Fundamentals of Carrier Transport*. (Cambridge University Press, 2009).
2. Cohen-Tannoudji, C., Diu, B. & Laloe, F. *Quantum Mechanics*. (Wiley, 1992).
3. Futterman, W., Osborne, E. & Saxon, D. S. A numerical solution of Schrodinger's equation in the continuum. *Journal of Research of the National Bureau of Standards* **52**, (1954).
4. Freysoldt, C. *et al.* First-principles calculations for point defects in solids. *Rev. Mod. Phys.* **86**, 253–305 (2014).
5. Kumagai, Y. & Oba, F. Electrostatics-based finite-size corrections for first-principles point defect calculations. *Phys. Rev. B* **89**, 195205 (2014).
6. Giannozzi, P. *et al.* QUANTUM ESPRESSO: a modular and open-source software project for quantum simulations of materials. *J. Phys.: Condens. Matter* **21**, 395502 (2009).
7. Perdew, J. P., Burke, K. & Ernzerhof, M. Generalized gradient approximation made simple. *Phys. Rev. Lett.* **77**, 3865–3868 (1996).
8. Baroni, S., de Gironcoli, S., Dal Corso, A. & Giannozzi, P. Phonons and related crystal properties from density-functional perturbation theory. *Rev. Mod. Phys.* **73**, 515–562 (2001).
9. Giustino, F., Cohen, M. & Louie, S. Electron-phonon interaction using Wannier functions. *Phys. Rev. B* **76**, 165108 (2007).
10. J. M. Ziman. *Electrons and Phonons: The Theory of Transport Phenomena in Solids*. (Clarendon Press, 1960).
11. Hellman, O., Steneteg, P., Abrikosov, I. A. & Simak, S. I. Temperature dependent effective potential method for accurate free energy calculations of solids. *Phys. Rev. B* **87**, 104111 (2013).
12. Tadano, T., Gohda, Y. & Tsuneyuki, S. Anharmonic force constants extracted from first-principles molecular dynamics: applications to heat transfer simulations. *J. Phys.: Condens. Matter* **26**, 225402 (2014).
13. Han, W. *et al.* Spin injection and detection in lanthanum- and niobium-doped $SrTiO_3$ using the Hanle technique. *Nat. Comm.* **4**, 2134 (2013).
14. Azadmanjiri, J. *et al.* A review on hybrid nanolaminate materials synthesized by deposition techniques for energy storage applications. *J. Mater. Chem. A* **2**, 3695–3708 (2014).
15. *Non-Tetrahedrally Bonded Elements and Binary Compounds I*. vol. 41C (Springer-Verlag, 1998).
16. J. Slade, T. *et al.* Understanding the thermally activated charge transport in $NaPb_mSbQ_{m+2}$ (Q = S, Se, Te) thermoelectrics: weak dielectric screening leads to grain boundary dominated charge carrier scattering. *Energy & Environmental Science* **13**, 1509–1518 (2020).
